# A Graph-Based Deep Learning Framework with Gating and Omics-Linked Attention for Multi-Omics Integration and Biomarker Discovery

**DOI:** 10.3390/biology14121764

**Published:** 2025-12-10

**Authors:** Zhanpeng Huang, Yutao Deng, Jinyuan Liu, Zhaohan Cai

**Affiliations:** College of Medical Information Engineering, Guangdong Pharmaceutical University, Guangzhou 510006, China

**Keywords:** multi-omics integration, deep learning, graph neural network, gating and confidence mechanisms, attention mechanism, biomarkers identification

## Abstract

Integration of multi-omics data provides a comprehensive perspective on complex biological systems, facilitating advances in disease classification and biomarker discovery. However, the heterogeneity and high dimensionality of omics data present significant analytical challenges. To achieve effective and interpretable multi-omics integration, we propose a novel deep learning framework named MOGOLA(Multi-Omics integration by Gating and Omics-Linked Attention). MOGOLA consists of three core components: (1) A hybrid graph learning module that integrates Graph Convolutional Networks and Graph Attention Networks for intra-omics feature extraction. (2) A gating and confidence mechanism that adaptively weighs feature importance across different omics types. (3) A cross-omics attention-based fusion module that captures inter-omics relationships. Comprehensive evaluations on four benchmark datasets (BRCA, KIPAN, ROSMAP, and LGG) demonstrate that MOGOLA consistently outperforms eleven state-of-the-art approaches. Ablation studies further validate the contribution of each module, while biomarkers identification highlight the framework’s clinical potential. These results show that MOGOLA is a robust and interpretable approach for multi-omics data integration and a contribution to advances in computational biology and precision medicine.

## Simple Summary

Understanding complex diseases like cancer and Alzheimer’s requires analyzing multiple types of biological data, including gene expression, DNA methylation, and microRNA. Integrating these multi-omics datasets provide a more comprehensive understanding of disease mechanisms. In this study, we present MOGOLA(Multi-Omics integration by Gating and Omics-Linked Attention), a novel deep learning framework that effectively integrates multi-omics data through graph-based learning and attention mechanisms. Our approach captures critical patterns within each omics data and uncovers meaningful relationships across them. Evaluations on several real-world disease datasets, including cancer and Alzheimer’s disease, demonstrate that MOGOLA outperforms existing methods in classification accuracy and successfully identifies key biomarkers with strong biological relevance.

## 1. Introduction

With the rapid development of sequencing technologies and high-throughput biomedical platforms has enabled the generation of increasingly comprehensive omics datasets, revolutionizing our ability to investigate biological systems [1]. By integrating these diverse data sources, researchers can perform deeper analyses that improve clinical decision making and advance disease treatment outcomes [2]. Consequently, numerous machine learning-based methods have been proposed for multi-omics data integration [3,4].

The integration of omics data can be broadly categorized into supervised and unsupervised approaches [5]. Unsupervised methods typically map multi-omics data into a unified low-dimensional space for clustering, as seen in multi-view clustering with low-rank and sparsity constraints [6] and decoupled contrastive clustering [7]. However, the scarcity of annotated labels limits their biological interpretability, motivating the adoption of supervised approaches. For example, the group-regularized ridge regression method has been proposed to adaptively integrate co-data by adjusting penalty intensities across variable groups [8]. Similarly, using latent components for data integration has led to sparse generalized canonical correlation analysis, which discriminates phenotypic groups and identifies shared information across omics modalities [9]. Recently, dynamic fusion frameworks such as Multi-Modality Dynamics (MMDynamic) have been introduced to improve both performance and trustworthiness by adaptively weighting informative features and modalities for each sample [10]. Despite these advancements, many of these methods rely on simplified linear assumptions that cannot fully capture the nonlinear complexities inherent in biomedical data.

Deep learning has emerged as a powerful solution for multi-omics integration due to its ability to model nonlinear relationships and learn modality-specific representations [11,12]. To reconstruct multi-omics data using such representation, Chen Zhao et al. proposed CLCLSA (Cross-omics Linked unified embedding with Contrastive Learning and Self Attention), a deep learning multi-omics integration framework [13]. In parallel, graph-based representation learning methods have demonstrated great potential in bioinformatics, as many biomedical entities and their interactions can be naturally encoded as graphs [14,15,16]. Graph Neural Networks and their variants utilize relational structures to model interactions among biomolecules, patients, or clinical variables, thereby improving both predictive performance and interpretability. For instance, MOGONET [17] integrates within-omics features using Graph Convolutional Networks (GCNs) [18] and employs correlation discovery for cross-omics integration. Similarly, MOSGAT [19] incorporates specificity-aware graph attention and cross-modal learning based on Graph Attention Networks (GATs), which enhances disease diagnosis through adaptive attention weighting and inter-omics feature discovery. These advances highlight the versatility and potential of graph-based frameworks, along with applications such as circRNA drug resistance prediction [20,21]. Moreover, attention mechanisms further strengthen feature fusion by allowing models to selectively focus on the most informative signals [22].

Despite these advances, several key challenges remain. Effective integration of multi-omics data requires extracting sample-specific features within each omics data while simultaneously identifying cross-omics correlations. Existing methods often fall to dynamically capture the varying information across omics for different samples, which undermines interpretability and limits the reliability of identified biomarkers [23]. Additionally, prior research has largely overlooked latent correlations within omics data, intuitively, data with similar structures should yield similar representations.

To address these challenges, we propose MOGOLA (Multi-Omics integration by Gating and Omics-Linked Attention), a novel framework for multi-omics integration and biomarker discovery. For each omics dataset, cosine similarity is computed between inter-omics samples, followed by the construction of a similarity matrix using a K-nearest-neighbors strategy with threshold-based filtering. Both the similarity matrix and the raw omics data are processed using graph structure learning modules. By combining the strengths of Graph Convolutional Networks (GCNs) and Graph Attention Networks (GATs), MOGOLA introduces gating and confidence mechanisms to enhance cross-omics feature selection. Furthermore, a novel cross-omics attention mechanism is incorporated to implicitly capture latent inter-omics correlations, thereby optimizing the fusion process and improving the identification of disease-associated biomarkers.

## 2. Materials and Methods

### 2.1. Overview of MOGOLA

**Figure 1:**
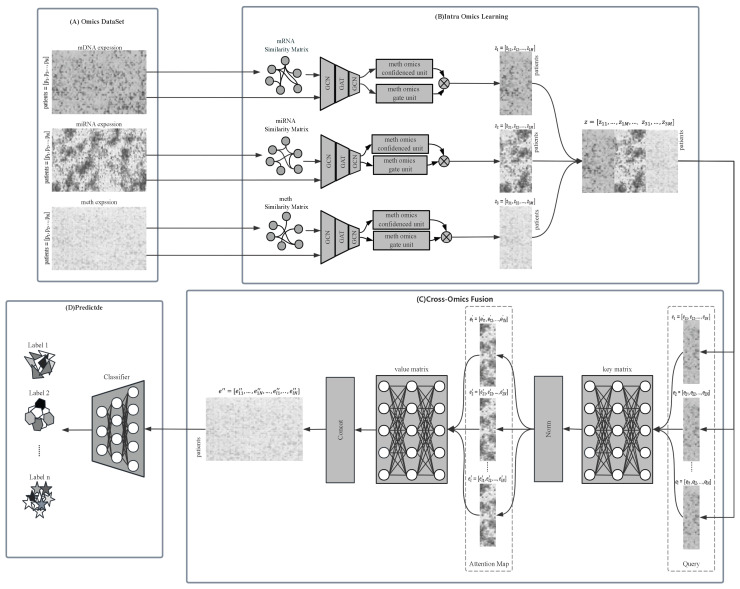
Framework of proposed MOGOLA.

MOGOLA is a two-stage framework designed to effectively learn from multi-omics information and capture its relationship with the ground-truth labels. As shown in Figure 1, the framework comprises two main components: Inter-Omics Learning and Cross-Omics Fusion.

In the Inter-Omics Learning stage, MOGOLA constructs omics specific graph structures to model sample relationships within each omics type. A confidence-weighted gating mechanism adaptively refines feature interactions across omics. In the Cross-Omics Fusion stage, an omics-linked attention mechanism integrates the heterogeneous omics representations obtained from each omics, allowing the model to focus on highly informative and complementary features across modalities. Detailed description of each component are provided in the subsequent sections.

### 2.2. Inter-Omics Learning

#### 2.2.1. Sample Similarity Matrix Construction

Constructing an accurate sample similarity matrix serves as the fundamental step for building the graph structure in MOGOLA. For each omics data, samples were first represented as feature vector, and pairwise similarity was quantified using cosine similarity. Given two samples *i* and *j* from the *m*-th omics, the cosine similarity is computed as follows:

(1) $$dist\left(x_{i}^{m},x_{j}^{m}\right)=\cos\frac{x_{i}^{m}\cdot x_{j}^{m}}{\|x_{i}^{m}{\|}_{2}\cdot {\left\|x_{j}^{m}\right\|}_{2}}$$

where $x_{i}^{m}$ and $x_{j}^{m}$ denote the *m*-th omics feature vectors of nodes *i* and node *j*, “·” represents the dot product operation, and “${∥\cdot ∥}_{2}$” denotes the Euclidean norm.

To construct a sparse and meaningful graph, a K-Nearest-Neighbors (KNN) [24] strategy was applied based on these similarity values. For each sample, cosine similarities were sorted in descending order, and the top *k* Nearest Neighbors were retained as connected edges. The resulting sample similarity matrix for the *m*-th omics is defined as follows:

(2) $$S^{m}=\left\{\begin{array}{cc} 1, & \mathrm{if}\;dist(x_{i}^{m},x_{j}^{m})>k\\ 0, & \mathrm{otherwise} \end{array}\right.$$

where $S^{m}$ denotes the binary similarity matrix corresponding to the *m*-th omics data, and *k* controls the sparsity level. All non-neighboring pairs are assigned zero similarity. This KNN-based matrix construction ensures that each graph maintains local structural information while filtering out weak or noisy relationships.

#### 2.2.2. Graph Structure Learning Module

To capture complex relationships with each omics, MOGOLA employs a graph structure learning module that integrates Graph Convolutional Network (GCN) [18] and the Graph Attention Network (GAT) [19]. This hybrid design extracts both local structural information, producing refined latent features.

The GCN layer aggregates features based on the similarity matrix $S^{m}$ and generates low-dimensional embedding. Given omics data $X^{m}$ and similarity matrix $S^{m}$, the GCN propagation rule is

(3) $$GCN\left(S^{m},X^{m}\right)=ReLU\left({\left(D^{m}\right)}^{-0.5}\left(S^{m}+I\right){\left(D^{m}\right)}^{-0.5}X^{m}W^{m}\right)$$

where $D^{m}$ is a diagonal matrix of $S^{m}+I$, I is the identity matrix, adding self-loops to the $S^{m}$. $W^{m}$ is a learnable weight matrix, and ReLU() is a nonlinear activation function. Through iterative message passing, nodes aggregate information from their neighbors to obtained enriched feature representations.

To overcome the limitations of uniform neighborhood aggregation in GCN, GAT assigns different weights to neighboring nodes via a self-attention mechanism. The GAT layer computes node embeddings as follows:

(4) $$GAT\left(S^{m},X^{m}\right)=ReLU\left(\sum_{j\in Nei(i)}\theta_{ij}^{m}W^{m}x_{j}^{m}\right)$$

where $W^{m}$ is a learnable weight matrix, and Nei denotes the set of neighbors of node *i*. The term $\theta_{ij}^{m}$ represents the attention coefficient between node *i* and node *j* in the *m*-th omics, which is computed as follows:

(5) $$\theta_{ij}^{m}=\frac{\exp\left(LR\left(a^{T}\cdot Concat\left(W^{m}x_{i}^{m},W^{m}x_{j}^{m}\right)\right)\right)}{\sum_{k\in Nei(i)}\exp\left(LR\left(a^{T}\cdot Concat\left(W^{m}x_{i}^{m},W^{m}x_{k}^{m}\right)\right)\right)}$$

where *a* denotes the learnable attention vector, LR() represents the Leaky Relu activation function, and Concat() indicates the concatenation operation applied to the transformed node features. Using the attention weights $\theta_{ij}^{m}$, GAT performs a weighted aggregation of the features from neighborhood nodes to obtain refined latent representation. By introducing dynamic, data-driven node relationship through attention mechanism, GAT enhances feature learning and captures the underlying graph structures.

To integrate the structural advantages of GCN with the adaptive learning capability of GAT, our module combines GCN’s structural stability with GAT’s dynamic attention mechanism. GCN aggregates information from fixed neighborhoods to capture local topological features and preserve graph stability, whereas GAT uses learnable attention weights to selectively emphasize informative neighbors, thereby enhancing global semantic relationships. Specifically, to address the common issues of over-smoothing in deep architectures and insufficient pattern extraction in shallow network, we adopt a three-layer design. The first GCN layer aggregates structured neighborhoods to capture high level topological features and establish a foundational embedding. The intermediate GAT layer then adaptively re-weights neighborhood contributions, suppressing noise while preserving salient features. The output was subsequently passed to a final GCN layer, which further consolidates the refined neighborhood information into the final latent representation. The hybrid representation learning process for the *m*-th omics is defined as follows:

(6) $$\left\{\begin{array}{cc} Z_{1}^{m}=GCN\left(S^{m},X^{m}\right) & \\ Z_{2}^{m}=GAT\left(S^{m},Z_{1}^{m}\right) & \\ Z^{m}=GCN\left(S^{m},Z_{2}^{m}\right) \end{array}\right.$$

where $Z_{1}^{m}$, $Z_{2}^{m}$, and $Z^{m}$ denote the *m*-th omics latent feature matrix, respectively. Alternating GCN and GAT layers enable the model to preserve graph structural consistency while adaptively enhancing biological marker aggregation.

During this stage, a separate similarity matrix and graph structure are constructed for each omics data. Each graph structure processes only its corresponding omics data, thereby preserving modality-specific characteristics and preventing cross-omics interference.

#### 2.2.3. Gating and Confidence Learning Mechanism

High-dimensional data often contain heterogeneous noise and variable feature relevance across modalities, which may degrade classification performance. To address these challenges, MOGOLA employs a gating mechanism for adaptive feature selection and a True-Class Probability (TCP) [25] technique to estimate the reliability of each omics data.

Given the latent embedding $Z^{m}$, a linear projection l(), followed by a nonlinear activation function, produces an intermediate representations. A gating function g() was then applied to derive the gated features:

(7) $${\tilde{Z}}_{\mathrm{gate}}^{m}=g\left(\delta \left(l\left(Z^{m}\right)\right)⊙Z^{m}\right)$$

where ${\tilde{Z}}_{\mathrm{gate}}^{m}$ represents the gating feature matrix for the *m*-th omics, δ() is the sigmoid activation function, and ⊙ denotes element-wise multiplication.

To further regulate the contribution of each omics, a TCP-based confidence mechanism is introduced. TCP evaluates the probability assigned to the true class for each sample, reducing confidence when misclassification occurs and thus reflecting uncertainty in the corresponding omics. These confidence scores are then incorporated as adaptive weights to prioritize more reliable modalities during fusion.

Specifically, we define a linear layer *t*() to compute the confidence score through encoding the $Z^{m}$ into a vector, from which the TCP weighted matrix ${\tilde{Z}}_{\mathrm{tcp}}^{m}$ is obtained as follows:

(8) $${\tilde{Z}}_{\mathrm{tcp}}^{m}=t\left(Z^{m}\right)⊙Z^{m}$$

The final latent representation $\tilde{Z}$ for *m*-th omics was obtained by combining gating-based and confidence-based refinements:

(9) $${\tilde{Z}}^{m}={\tilde{Z}}_{\mathrm{gate}}^{m}⊙{\tilde{Z}}_{\mathrm{tcp}}^{m}$$

During the inter-omics learning stage, the graph structure learning module extracts latent structure features, while the gating and confidence mechanisms refine and adaptively weigh these representations. Together, these components enable MOGOLA to capture modality-specific patterns, suppress noise, and enhance discriminative learning across heterogeneous omics datasets.

### 2.3. Omics-Linked Attention for Cross-Omics Fusion

Based on the previously obtained latent representations for each omics, we introduce the Omics-Linked Attention (OLA) mechanism [26] to achieve cross-omics feature fusion and final prediction. Unlike traditional self-attention, which only considers single-omics samples and incurs high computational complexity, OLA captures cross-omics relationships by computing attention across different omics data.

Specifically, before computing OLA, the omics features ${\tilde{Z}}^{m}$ are concatenated to form a combined omics feature matrix $\bar{Z}$. For the *i*-th node in $\bar{Z}$, its similarity with the *j*-th row of the omics link unit matrix $U_{\mathrm{key}}$ is used to compute the attention score matrix A, as follows:

(10) $$\bar{Z}=\mathrm{Concat}\left({\tilde{Z}}^{1},\ldots ,\;{\tilde{Z}}^{m}\right)$$

(11) $$A=Norm(\bar{Z}U_{key})$$

where $U_{key}$ is a learnable weight matrix that serves as a memory of the entire training dataset, calculating similarity with input features to generate the attention map A. This attention map represents the similarity between the *i*-th node of $\bar{Z}$ and the *j*-th row of $U_{key}$. To eliminate sensitivity to input scale, the output is normalized twice-first across columns using softmax, then across rows using L1-normalization, which is formulated as

(12) $$\hat{A}=\bar{Z}U_{key}$$

(13) $${\left(\overset{˘}{A}\right)}_{ij}=\frac{\exp{\left(\hat{A}\right)}_{ij}}{\sum_{l=0}^{N}\exp{\left(\hat{A}\right)}_{lj}}$$

(14) $${\left(A\right)}_{ij}=\frac{{\left(\overset{ˇ}{A}\right)}_{ij}}{\sum_{p=0}^{d}{\left(\overset{ˇ}{A}\right)}_{ip}}$$

where $\hat{A}$ and $\overset{˘}{A}$ are separately normalized columns and rows.

Each normalization has a computational complexity of O(N), where *N* is the number of nodes, ensuring minimal overhead and preserving scalability even for large graphs. Finally, the features are updated based on the attention scores in A, producing the output Y:

(15) $$Y=AU_{val}$$

where $U_{val}$ reconstructs the output by transforming the attention map A into enhanced features using optimized prototypes from the entire dataset. As described above, the complete calculation of OLA can be expressed as

(16) $$Y=Norm\left(\bar{Z}U_{key}\right)U_{val}$$

During multi-head attention computation, conventional self-attention splits input features into several blocks, computes attention independently within each block, and then concatenates the results. While this reduces computation and memory usage, it may degrade performance. In contrast, OLA allows all tokens to interact via omics link $U_{key}$ and $U_{val}$, improving performance while reducing the amount of parameter. Multi-head OLA is implemented as follows:

(17) $$z_{1},\ldots ,z_{e},\ldots ,z_{H}=Split\left(\bar{Z}\right)$$

(18) $$Y_{e}=Norm\left(z_{e}U_{key}\right)U_{val}$$

(19) $$\bar{Y}=Concat\left(Y_{1},Y_{2},...,Y_{H}\right)U_{t}$$

where Split() divided input features into several blocks, $z_{e}$ and $Y_{e}$ denotes the *e*-th feature block and attention head, respectively, and *H* is the total number of heads. $U_{key}$ and $U_{val}$ correspond to different heads, and $U_{t}$ is a transformation matrix ensuring consistency between the input and output feature dimensions. The resulting $\bar{Y}$ is a feature matrix weighted by OLA.

Compared with standard self-attention, OLA is more concise and computationally efficient, enhancing model performance while significantly reducing parameter count. The fused multi-omics feature matrix $\bar{Y}$ is subsequently passed through a fully connected layer, whose output serves as the final prediction of the model.

### 2.4. Model Optimization

To improve the model performance and training stability, MOGOLA adopts a two-step learning strategy. The first stage focuses on optimizing the omics-specific graph structures, while the second stage jointly optimizes the entire network, including the gating and confidence modules.

#### 2.4.1. Graph Structure Optimization

In this stage, the latent features generated by the graph structure learning module are fed into a classifier to predict sample labels. The model is trained using a supervised loss function defined as

(20) $$L_{\mathrm{GS}}=-\sum_{m=1}^{M}\sum_{n=1}^{N}log\left(\frac{e^{{\hat{y}}_{n}^{m}\cdot y_{n}}}{\sum_{q=1}^{Q}e^{{\hat{y}}_{n,q}^{m}}}\right)$$

where $y_{n}$ denotes the true label of the *n*-th sample, and ${\hat{y}}_{n,q}^{m}$ represents the predicted probability that the *n*-th sample feature in the *m*-th omics belongs to class *q*. The terms *M* and *N* indicate the number of omics type and the number of samples. In addition, class-specific loss weights are incorporated to address label imbalance in the training set.

#### 2.4.2. Gating and Confidence Learning Optimization

In the second stage, we optimize the gating and confidence module by minimizing the loss functions corresponding to their respective tasks.

First, given the latent omics representation $Z^{m}$, the classifier c() followed by a softmax() operator produces a confidence-based predictive distribution:

(21) $$\rho_{n}^{m}=softmax\left(c\left(Z^{m}\right)\right)$$

where $\rho_{n}^{m}$ is the predicted probability from the classifier corresponding to the *m*-th omics.

Next, the classifier confidence for each omics is optimized using the cross-entropy loss:

(22) $$L_{ce}=-\sum_{m=1}^{M}\sum_{n=1}^{N}y_{n}log\rho_{n}^{m}$$

where $y_{n}$ is the ground-truth label of the *n*-th sample.

Additionally, based on predicted distribution $\rho_{n}^{m}$ and the true label $y_{n}$, the confidence score ${TCP}^{m}$ is computed as follows:

(23) $$TCP^{m}=\sum_{n=1}^{N}y_{n}{\hat{p}}_{n}^{m}$$

To train the confidence estimator $t(Z^{m})$, the MSE loss is defined as follows:

(24) $$L_{mse}=\sum_{m=1}^{M}{\left(t\left(Z^{m}\right)-TCP^{m}\right)}^{2}$$

Finally, the overall gating and confidence loss functions $L_{GC}$ is given by

(25) $$L_{GC}=L_{ce}+L_{mse}$$

#### 2.4.3. Final Objective

The final loss function of MOGOLA integrates all optimization components and is defined as follows:

(26) $$L_{total}=L_{GS}+{\lambda_{1}L}_{GC}+{\lambda_{2}L}_{OLA}$$

where $L_{OLA}$ is the cross-entropy loss computed from final output of the Omics-Linked Attention (OLA) mechanism. The hyperparameters $\lambda_{1}$ and $\lambda_{2}$ are used to balance the contribution of each component.

During training, we first pretrain each omics-specific graph structure using the graph structure loss $L_{GS}$. Then, the omics-specific graph structure parameters are frozen, and the remaining components—including gating, confidence estimation, and the OLA module—are jointly optimized to minimize $L_{total}$. This two-stage training scheme enables the model to first learn robust graph-based representations and then refine inter-omics fusion through confidence-aware learning, ultimately improving prediction accuracy and biological interpretability.

## 3. Results

To evaluate the performance of the proposed MOGOLA framework, we conducted a comprehensive set of experiments on four publicly available multi-omics datasets.

### 3.1. Data Sources

We employed four public available multi-omics datasets to evaluate the performance of MOGOLA: BRCA (breast cancer) [27], KIPAN (kidney cancer) [28], ROSMAP (Alzheimer’s disease) [29], and LGG (lower-grade glioma) [30]. Each dataset contains three omics: mRNA expression (mRNA), DNA methylation (meth), and miRNA expression (miRNA). Specifically, the BRCA dataset is used for PAM50-subtype classification of breast invasive carcinoma, KIPAN for kidney cancer type classification, ROSMAP for Alzheimer’s disease patient versus normal control classification, and LGG for glioma grading.

During the preprocessing stage, we performed systematic feature refinement for each omics following the procedure described in [17]. Detailed statistics and feature counts are summarized in Table 1.

### 3.2. Loss Function Hyperparameter Experiment

For loss balancing coefficients, the hyperparameters $\lambda_{1}$ and $\lambda_{2}$ control the relative contributions of different loss components during training. We perform a grid search strategy to determine suitable values for $\lambda_{1}$ and $\lambda_{2}$, evaluating all combinations selected from {0.01, 0.1, 1, 10, 100}.

**Figure 2:**
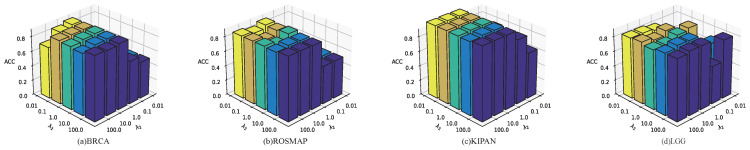
Hyperparameter evaluation for loss function.

As shown in Figure 2, the optimal hyperparameter vary across datasets. When both $\lambda_{1}$ and $\lambda_{2}$ are set to 10, MOGOLA achieves the best performance on the LGG dataset. For all other datasets, MOGOLA gains optimal performance with $\lambda_{1}$ =1 and $\lambda_{2}$ = 1. These experimental results demonstrate that appropriately weighting each loss component contributes to improved overall algorithm performance.

### 3.3. Evaluation and Compared with State-of-Art Methods

For multi-class tasks, we used the following evaluation metrics: accuracy rate (ACC), weighted F1 score (F1_weighted), and macro-averaged F1 score (F1_score). For the binary classification task, we employed the accuracy rate (ACC), F1 score (F1), and the area under the receiver operating characteristic curve (AUC) for performance evaluation.

For each dataset, 30% of samples were randomly selected as the test set, while the remaining 70% were used for training. The test samples were stratified to preserve the original class distribution. All methods were evaluated using five independent random splits, and the mean ± standard deviation of each metrics was reported to ensure statistical reliability.

Since each dataset exhibits distinct topological characteristics, different hyperparameters were selected accordingly. The hyperparameter *k* determines the number of nearest neighbors retained for each sample. A smaller *k* yields a sparser graph and may miss important relationships, while a larger *k* results in a denser graph that may introduce noise. We tested values of *k* from 2 to 10 and selected the optimal values for each dataset. The final *k* values were set to 9, 2, 3, and 7 for the BRCA, ROSMAP, KIPAN, LGG datasets, respectively.

We compared MOGOLA with eleven state-of-the-art classification approaches, including traditional machine learning, deep learning, and multi-omics integration models: Lasso regression (Lasso) [31], XGBoost [32], Fully-Connected NN (FCNN) [33], BSPLSDA [9], Trusted Multi-View Classification (TMC) [34], Cascade of Final Multimodal Representations (CF) [35] Gated Multimodal Unit (GMU) [36], MOGONET [17], MMDynamic [37], CLCLSA [13] and MOSGAT [38].

As summarized in Table 2 and Table 3, MOGOLA consistently outperforms all baseline methods across both binary and multi-class classification tasks. It achieves the highest accuracy, F1-score, and AUC on all four datasets, demonstrating its superior capability in integrating and interpreting heterogeneous omics data. For the KIPAN dataset, where subtype distinctions are more pronounced, most methods achieve near-perfect performance. Nevertheless, MOGOLA still matched or slightly exceeded the compared methods, highlighting its stability and robustness on multi-classification datasets.

These results indicate that MOGOLA not only maintains strong generalization ability but also provides significant performance gains, particularly on complex multi-classification tasks such as BRCA and LGG.

### 3.4. Model Performance with Different Omics Data Types

To evaluate the necessity of integrating multi-omics data, we performed experiments using different combinations of omics types on the BRCA, ROSMAP, KIPAN, and LGG datasets. Specifically, we compared the performance of MOGOLA when integrating all three omics (miRNA + mRNA + methylation) and when using pairwise combinations (miRNA + mRNA, mRNA + methylation, miRNA + methylation). All experimental settings were consistent with those used in the comparative experiments.

**Figure 3:**
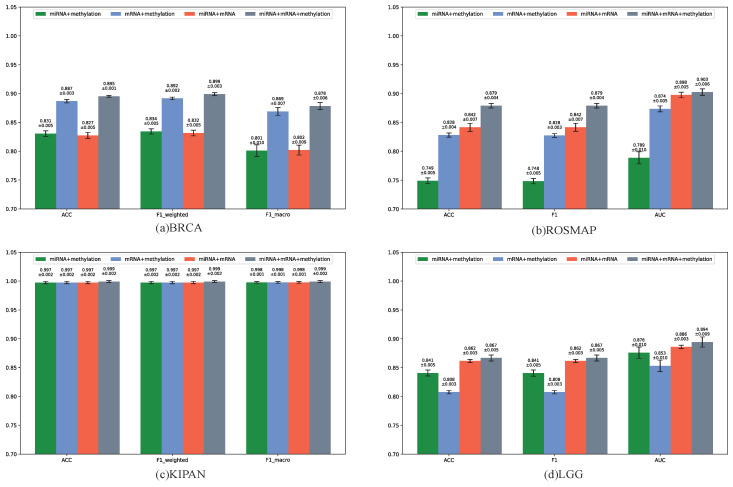
Performance comparison across different omics combinations.

As shown in Figure 3, integrating all three omics consistently produced the best classification performance, demonstrating the importance of comprehensive multi-omics integration. To further confirm this, we compared three-omics integration with two-omics integration (Supplementary Material Table S1). Across all datasets, MOGOLA consistently outperformed competing methods in most classification tasks. These findings highlight the effectiveness of graph structure learning for omics data classification and the strong potential of cross-omics learning through the proposed Omics-Linked Attention mechanism in enhancing biomedical data analysis.

### 3.5. Ablation Analysis

To assess the individual contributions of the MOGOLA’s main components, we conducted a comprehensive ablation study focusing on three key modules: the Graph Structure Learning (GSL) module, the confidence-based Feature Gate module, and the cross-omics Attention (Omics-Linked Attention) module. For ablated models, the GSL module was replaced with a linear layer, the gating mechanism was removed, and the Omics Attention module was bypassed by directly concatenating processed features before classification. Experiments were conducted on all four datasets.

As summarized in Table 4 and Supplementary Material Table S2, removing or replacing any module resulted in a noticeable performance decline, confirming the essential role of each component. In particular, replacing the hybrid graph structure with a linear layer led to a substantial accuracy drop, demonstrating that graph structure learning effectively captures sample similarities and enhances multi-omics integration.

To further validate the design of the hybrid graph structure, we tested different combinations of GCN and GAT layers. As shown in Table 5 and Supplementary Material Table S3, the GCN-GAT-GCN structure achieved the best performance. This architecture combines GCN’s neighborhood smoothing and GAT’s adaptive neighbor weighting. By alternating GCN and GAT layers, MOGOLA preserves structural information in omics data while enhancing key neighbor aggregation. Compared to pure GCN or GAT architectures, this hierarchical design gains a strong balance between computational efficiency and classification accuracy.

We also evaluated different depths of alternating GCN and GAT layers (Supplementary Material Table S4). The three-layer architecture achieved the best performance, confirming that it strikes an effective balance between smoothing and expressive feature extraction. Excessively deep or shallow networks degraded performance due to over-smoothing or insufficient pattern learning.

Finally, we assess the effectiveness of MOGOLA’s cross-omics fusion strategy by replacing the graph attention module with alternative fusion mechanisms, including standard self-attention module and a view-correlation discovery network (VCDN) [17]. As shown in Table 6 and Supplementary Materials Table S5, MOGOLA’s graph attention-based fusion consistently outperformed these alternatives. This demonstrates that OLA effectively captures latent functional associations across different omics, whereas VCDN and self-attention offer comparatively limited improvements.

Overall, these results demonstrate that the three primary modules of MOGOLA is critical for capturing multi-omics interactions and achieving robust classification performance, including graph structure learning, confidence-based gating, and cross-omics attention.

## 4. Biomarker Discovery and Analysis of BRCA

Biomarkers serve as measurable molecular indicators of disease risk, occurrence, and patient prognosis. Accurate identification of biomarkers not only enhances early disease diagnosis and risk assessment but also improves personalized treatment and drug discovery. Moreover, biomarkers discovery is essential for model interpretability and understanding molecular mechanisms [39].

### 4.1. Biomarker Discovery of BRCA

In this study, we conducted biomarkers identification analysis on the BRCA dataset. For each sample in the test set, individual features were sequentially masked (set to zero), and the trained MOGOLA model was used to reclassify the modified inputs data. The macro-F1 score was computed as the evaluation metrics. By comparing the performance drops between the complete dataset and each feature-ablated version, we quantified the relative importance of every feature [40]. MOGOLA has identified 464 mRNA biomarkers, 294 methylation biomarkers, and 136 miRNA biomarkers. Their names and importance scores are provided in the Supplementary Material Table S6. Table 7 presents only the top 10 most important biomarkers identified from each omics type.

Several mRNA biomarkers identified in BRCA have been previously implicated in breast cancer progression. C1orf106 [41] is frequently amplified and overexpressed in basal-like breast cancer. Similarly, SOX11 [42] is highly expressed in HER2-positive and basal-like subtypes and is strongly associated with recurrence and progression from ductal carcinoma in situ to invasive carcinoma. HPDL [43], a gene involved in tyrosine metabolism, has been linked to poor prognosis when overexpressed in breast cancer patients.

For DNA methylation-based biomarkers, SOX21 [44] promotes tumor proliferation, metastasis, and chemoresistance through the PI3K/AKT pathway and the miR-520a-5p/ORMDL3 regulatory axis. Its dysregulation correlates with advanced disease stage, lymph node metastasis, and poor survival. KEGG annotations also reveal several olfactory receptor genes (e.g., OR11H6, OR1J4, OR4N5, OR11G20) [44]. They are expressed in peripheral tissues and may have broader functions despite their canonical sensory roles. Additionally, ATP10B [45] is identified as a driver genes associated with high proliferation and recurrence risk in Luminal B-type breast cancer.

Among miRNA biomarkers, hsa-mir-210 [46] is upregulated under hypoxic conditions through the HIF-1α/VHL pathway and is independently associated with poorer survival outcomes. Hsa-mir-1307 [47] is significantly overexpressed in tumor tissues compared with adjacent normal tissues and strongly correlated with unfavorable prognosis. Hsa-mir-2355 [48] promotes immune evasion and chemoresistance in triple-negative breast cancer by interacting with NEAT1 lncRNA to elevate MSLN expression.

### 4.2. Pathway and Process Enrichment Analysis

To investigate the biological roles of the identified genes, we conducted pathway and process enrichment analysis using Gene Ontology (GO) Biological Processes and Kyoto Encyclopedia of Genes and Genomes (KEGG) pathways through the Metascape platform [49]. Analyses were performed using all genome-wide genes as the background. Only terms with *p*-value < 0.01, minimum count ≥ 3, and enrichment factor > 1.5 were retained.

Terms were clustered based on membership similarity using the Kappa statistic (threshold = 0.3). For each cluster, the most statistically significant term was selected to represent the cluster. *p*-values were computed using the cumulative hypergeometric distribution, and q-values were generated using the Benjamini–Hochberg correction.

**Figure 4:**
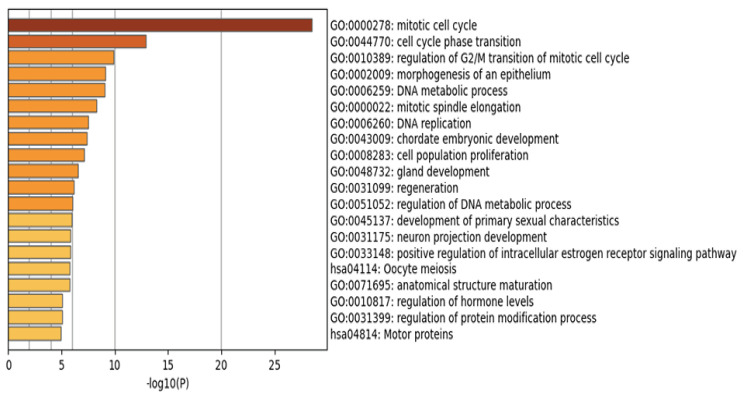
Enriched GO and KEGG pathways across genes colored by *p*-values.

As shown in Figure 4 and Supplementary Materials Table S7, the identified genes are involved in multiple biological pathways, consistent with previous studies. For example, breast cancer development is closely associated with dysregulation of the mitotic cell cycle pathway, a key mechanism driving malignant proliferation [50]. Additionally, abnormal regulation of epithelial morphogenesis contributes to the initiation and progression of breast cancer [51].

**Figure 5:**
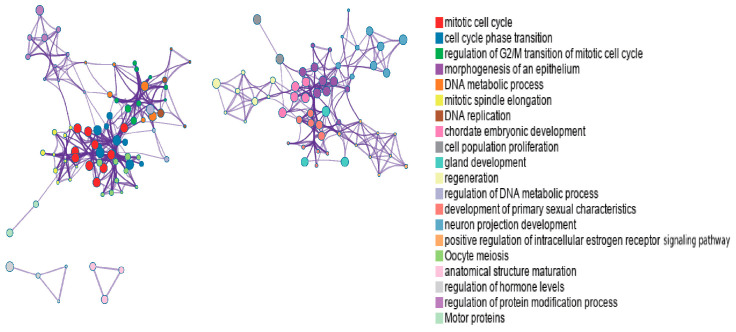
Network of enriched terms colored by cluster ID.

**Figure 6:**
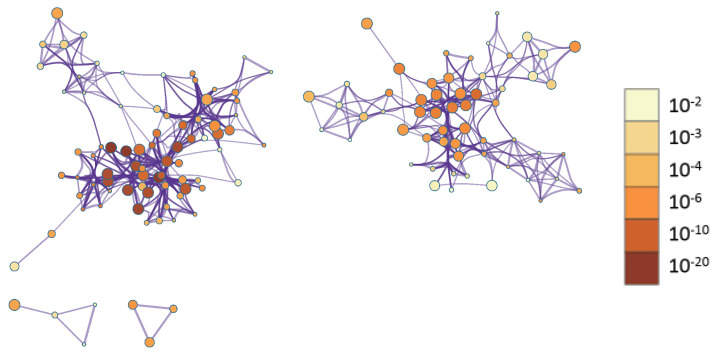
Network of enriched terms colored by *p*-value.

To better visualize relationships between enriched terms, a subset of terms was represented as a network, where edges indicate similarity > 0.3. We selected top-ranked terms from 20 clusters (up to 15 terms per cluster, capped at 250 terms total). Visualization in Cytoscape [52] colored nodes by cluster ID (Figure 5) and by *p*-value (Figure 6). In Figure 5, nodes sharing the same cluster ID are typically positioned close together, while in Figure 6, terms containing more genes tend to have more significant *p*-values.

### 4.3. Protein–Protein Interaction Analysis and Hub Gene Identification

To identify key mRNAs with potential functional relevance, we constructed a Protein–Protein Interaction (PPI) network using STRING [53] with a high-confidence threshold (0.9). The resulting network (Supplementary Materials Table S8) was imported into Cytoscape for hub gene analysis using CytoHubba, with detailed results presented in the Supplementary Materials Table S9. Hub genes were determined based on degree centrality, stress centrality, and betweenness centrality. Nineteen hub genes were identified: HJURP, TROAP, BYSL, CDC20, NOP58, UBE2C, POP1, CBX2, CCT4, CDC6, PLK1, CCNB2, CCNB1, BUB1, BIRC5, CDCA8, KIF2C, TPX2, and AURKB.

Among these, HJURP [54] is highly expressed in breast cancer and promotes tumor progression by stabilizing the YAP1 protein and activating transcription of NDRG1. BUB1 [55] and BYSL [56] are overexpressed in various solid tumors, including breast cancer, and are associated with poor prognosis. AURKB [57] promotes malignant proliferation of breast cancer cells through its protein stability and is a key driver of breast cancer development. KIF2C [58] is upregulated in breast cancer and may contribute to disease progression by promoting cell proliferation and migration.

Collectively, these hub genes identified by MOGOLA are well-documented, underscoring their clinical relevance and potential to guide prognostic assessment and therapeutic targeting.

### 4.4. Drug–Gene Interaction Analysis

**Figure 7:**
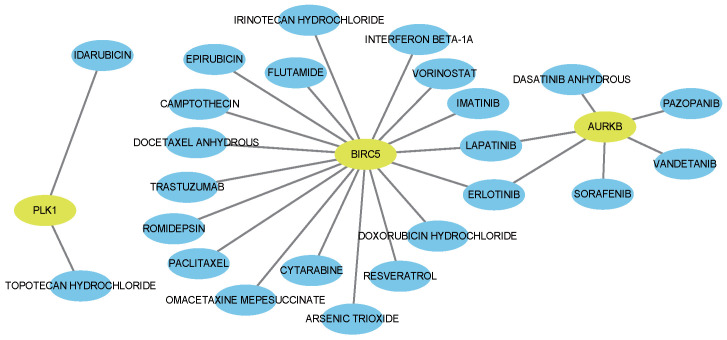
Network of drug–gene interactions (blue = drugs, yellow = genes).

Using the Drug–Gene Interaction Database (DGIdb) [59], we identified FDA-approved agents associated with the hub genes. This analysis yielded 69 drug–gene interactions (Supplementary Materials Table S10), including 24 antieoplastic agents (visualized in Figure 7). Notably, eight correspond to drugs already used clinically for breast cancer.

Anthracyclines (e.g., epirubicin, doxorubicin) remain core components of systemic therapy, functioning via DNA intercalation and free-radical generation [60]. Taxanes (docetaxel, paclitaxel) stabilize microtubules and are central to neoadjuvant, adjuvant, and metastatic treatment [61]. For patients resistant to anthracyclines or taxanes, irinotecan—particularly in liposomal form—has shown clinical activity, with reported ORRs ranging from 14–23% [62] and up to 34.5% in heavily pretreated patients [63].

In HER2-positive disease, trastuzumab greatly improves PFS and OS, while lapatinib demonstrates activity in trastuzumab-resistant cases, especially in combination regimens [64].

Epigenetic modulators such as vorinostat (SAHA) also show promise. When combined with tamoxifen, vorinostat achieved a clinical benefit rate of 40% in endocrine-resistant ER+ metastatic breast cancer [65]. When combined with paclitaxel and bevacizumab, it achieved an ORR of 55% with manageable toxicity [66]. Mechanistically, vorinostat enhances chemosensitivity by destabilizing Hsp90 client proteins [67] and can suppress proliferation in triple-negative breast cancer [57].

## 5. Discussion

High-throughput techniques have produced vast labeled multi-omics datasets, and integrated multi-omics approaches consistently outperform single-omics analyses in disease prediction [27,68]. In response to this challenge, we propose MOGOLA, a supervised multi-omics integration method that combines Graph Convolution Networks and Graph Attention Networks for inter-omics representation learning and employs gating and confidence mechanisms to enhance feature selection. This design effectively captures relationships across samples and omics layers. Additionally, we incorporate the Omics-Linked Attention mechanism for fusion across omics types, which reduces computational costs, extracts representative omics-specific features, and uncovers potential cross-omics links.

MOGOLA has been evaluated on cancer subtype datasets and neuropathy grading tasks, achieving superior performance compared with existing models. Additionally, experiments using different omics combinations confirm the importance of integrating multiple omics data types in biomedical applications. Furthermore, visualization of learned embeddings demonstrates clear separation of biological subtypes, reflecting the strong representation capability of MOGOLA. Ablation studies further validate the contributions of the hybrid graph structure, gating and confidence modules, and the Omics-Linked Attention mechanism, with the hybrid graph structure playing a particularly crucial role.

Importantly, MOGOLA identified biologically meaningful biomarkers for each omics type in an end-to-end manner without the need for additional feature selection procedures. In BRCA, the identified biomarkers correspond to clinically relevant pathways, key biological processes, and actionable drug targets, supporting subtype-specific therapeutic strategies such as HER2-targeted treatments.

MOGOLA not only outperforms other models but also provides strong biological interpretability. It highlights dysregulated proliferation, with enrichment in cell cycle and PI3K–Akt signaling as signaling pathways, while PPI analysis identified key hub genes (e.g., CCNA2, CDK1, ESR1). These results align with caner hallmarks and known drug targets, showing that MOGOLA captures disease-driving mechanisms rather than just correlations.

Let *m* be the number of omics, *N* the number of samples, and *d* the number of features. The time complexity of MOGOLA arises from three stages: (1) Graph structure, using two GCN layers and one GAT layer, with complexity of $O(N^{2}\times H+N\times d\times H)$, where *H* is the maximum hidden dimension. (2) Gating and confidence mechanism, with three linear layers, $O(m\times N\times d^{2}+N\times m\times d\times H)$. (3) Omics fusion and classification, with three linear layers and double normalization, $O(N\times m^{2}\times d^{2}/h+N\times d\times C)$, where *h* is the number of the head and *C* the number of classes. Overall, the complexity of MOGOLA is $O(m\times N^{2}\times H)$, roughly comparable to MOSGAT and slightly higher than MOGONET.

For parameter complexity analysis, MOGOLA includes a moderate GAT layer, involving fewer parameters than the GAT-heavy MOSGAT design. It has slightly more parameters than the GCN-only MOGONET. The Omics-Linked Attention mechanism introduces less overhead than MOSGAT’s full self-attention module and slightly more than MOGONET’s simple fully connected fusion. Despite this modest increase, MOGOLA delivers substantially better predictive performance.

Although MOGOLA achieves good performance on four test datasets, the algorithm still requires further optimization. The static sample similarity matrix may limit model accuracy, emphasizing the need for dynamic adjustment. In addition, MOGOLA relies on a fixed set from a public dataset, which may introduce substantial non-biological noise when applied to other datasets due to variations in data processing. Such noise can obscure true biological signals and degrade performance, making precise data preprocessing a key future challenge. Moreover, MOGOLA is designed for multi-omics data integration and analysis, and its modular architecture holds promise for extension to single-cell multi-omics data. Exploring these extensions could significantly expand the applicability of our framework to emerging single-cell omics datasets.

## 6. Conclusions

In this study, we introduced MOGOLA, a supervised multi-omics integration framework designed to analyze increasingly large and complex annotated datasets. By using hybrid graph structure learning, a gating and confidence mechanism, and the Omics-Linked Attention mechanism, MOGOLA effectively extract representative features and reveals potential interaction across diverse omics layers. Extensive experiment evaluations demonstrate the effectiveness and robustness of the method, and biomarker analyses further confirm the biological relevance of the identified features, highlight key network hubs, and reveal actionable therapeutic targets. Moving forward, future work will focus on optimizing the fusion and graph modules and enhancing robustness to data heterogeneity.

## Figures and Tables

**Table 1:** Summary of datasets.

Dataset	Categories	Features (mRNA, meth, miRNA) Before Processing	Features (mRNA, meth, miRNA) After Processing
BRCA	Normal-like:115, Basal-like:131, HER2-enriched:46, Luminal A:436, Luminal B:147	20531, 20106, 503	1000, 1000, 503
ROSMAP	NC:169, AD:182	55889, 23788, 309	200, 200, 200
KIPAN	KICH:66, KIRC:318, KIRP:274	20531, 20111, 445	2000, 2000, 445
LGG	Grade 2:246, Grade 3:264	20531, 20114, 548	2000, 2000, 548

**Table 2:** Results on multi-classification datasets.

Method	BRCA			KIPAN		
	ACC	F1_weighted	F1_macro	ACC	F1_weighted	**F1_macro**
Lasso	0.746±0.014	0.724±0.026	0.664±0.023	0.978±0.004	0.978±0.004	0.975±0.006
XGBOOST	0.782±0.006	0.769±0.013	0.709±0.023	0.993±0.008	0.993±0.008	0.989±0.014
FCNN	0.765±0.016	0.756±0.004	0.688±0.007	0.991±0.005	0.991±0.005	0.991±0.005
BSPLSDA	0.639±0.008	0.522±0.016	0.451±0.023	0.919±0.012	0.918±0.013	0.895±0.014
CF	0.820±0.018	0.828±0.019	0.790±0.015	0.992±0.005	0.992±0.005	0.988±0.009
TMC	0.843±0.015	0.842±0.019	0.816±0.029	0.997±0.003	0.997±0.003	0.994±0.005
GMU	0.811±0.049	0.807±0.018	0.779±0.018	0.977±0.016	0.976±0.017	0.958±0.032
MOGONET	0.821±0.007	0.813±0.007	0.776±0.007	0.997±0.005	0.997±0.005	0.995±0.010
MMDynamics	0.865±0.007	0.868±0.007	0.832±0.011	0.999±0.002	0.999±0.002	0.999±0.002
CLCLSA	0.848±0.010	0.845±0.015	0.772±0.037	0.999±0.002	0.999±0.002	0.999±0.002
MOSGAT	0.873±0.004	0.878±0.003	0.847±0.005	0.999±0.002	0.999±0.002	0.999±0.002
Ours	0.895±0.001	0.899±0.003	0.878±0.006	0.999±0.002	0.999±0.002	0.999±0.002

**Table 3:** Results on the binary classification datasets.

Method	ROSMAP			LGG		
	ACC	F1	AUC	ACC	F1	ACC
Lasso	0.740±0.041	0.777±0.049	0.766±0.005	0.770±0.015	0.777±0.018	0.817±0.030
XGBOOST	0.775±0.012	0.789±0.018	0.809±0.029	0.760±0.050	0.766±0.054	0.832±0.051
FCNN	0.755±0.021	0.764±0.021	0.827±0.025	0.734±0.023	0.758±0.024	0.817±0.037
BSPLSDA	0.733±0.033	0.754±0.015	0.840±0.028	0.685±0.025	0.662±0.030	0.730±0.026
CF	0.784±0.011	0.799±0.002	0.882±0.055	0.831±0.012	0.856±0.002	0.887±0.003
TMC	0.888±0.021	0.829±0.016	0.890±0.026	0.820±0.018	0.809±0.003	0.871±0.004
GMU	0.776±0.029	0.776±0.016	0.867±0.006	0.801±0.002	0.799±0.009	0.856±0.013
MOGONET	0.825±0.004	0.821±0.006	0.882±0.006	0.829±0.012	0.826±0.013	0.848±0.009
MMDynamics	0.821±0.018	0.827±0.015	0.901±0.004	0.822±0.011	0.824±0.007	0.862±0.003
CLCLSA	0.824±0.008	0.831±0.009	0.901±0.007	0.839±0.003	0.843±0.004	0.893±0.005
MOSGAT	0.830±0.012	0.827±0.013	0.873±0.011	0.824±0.007	0.823±0.007	0.857±0.013
Ours	0.879±0.004	0.879±0.004	0.903±0.006	0.867±0.005	0.867±0.005	0.894±0.009

**Table 4:** Ablation experimental results.

Method	BRCA			ROSMAP		
	ACC	F1_weighted	F1_macro	ACC	F1	AUC
No GSL	0.850±0.009	0.852±0.011	0.820±0.017	0.784±0.152	0.857±0.006	0.867±0.008
No Feature Gate	0.888±0.002	0.892±0.001	0.875±0.004	0.862±0.010	0.862±0.010	0.905±0.008
No Omics Attention	0.881±0.004	0.886±0.005	0.862±0.009	0.847±0.007	0.847±0.007	0.879±0.008
No Ablation	0.895±0.001	0.899±0.003	0.878±0.006	0.879±0.004	0.879±0.004	0.903±0.006

**Table 5:** Performance of different graph structure configurations.

Method	BRCA			ROSMAP		
	ACC	F1_weighted	F1_macro	ACC	F1	AUC
GAT-GAT-GCN	0.888±0.005	0.892±0.005	0.870±0.007	0.855±0.011	0.854±0.011	0.890±0.010
GAT-GCN-GAT	0.885±0.009	0889±0.010	0.866±0.014	0.849±0.016	0.849±0.016	0.904±0.015
GAT-GCN-GCN	0.888±0.005	0.895±0.002	0.873±0.012	0.845±0.013	0.845±0.013	0.896±0.010
GCN-GAT-GAT	0.889±0.008	0.892±0.007	0.868±0.013	0.856±0.012	0.856±0.012	0.904±0.011
GCN-GAT-GCN	0.895±0.001	0.899±0.003	0.878±0.006	0.879±0.004	0.879±0.004	0.903±0.006

**Table 6:** Cross-omics fusion mechanism comparison.

Method	BRCA			ROSMAP		
	ACC	F1_weighted	F1_macro	ACC	F1	AUC
Replace VCDN	0.872±0.003	0.873±0.005	0.832±0.010	0.860±0.009	0.860±0.009	0.896±0.014
Replace SA	0.885±0.005	0.889±0.005	0.865±0.009	0.865±0.003	0.865±0.003	0.889±0.004
No Ablation	0.895±0.001	0.899±0.003	0.878±0.006	0.879±0.004	0.879±0.004	0.903±0.006

**Table 7:** Top 10 biomarkers identified for BRCA.

Omics Type	Biomarkers
mRNA	C1orf106, SOX11, ABCC11, HPDL, NXN, FAM174B, DEGS2, SEC16A, CYP4B1, CYP4Z2P
DNA methylation	TMEM207, TM4SF18, SOX21, OR1J4, ATP10B, MIR563, KRTAP3-3, LOC100130264, CDH26, ROPN1
miRNA	hsa-mir-1180, hsa-mir-149, hsa-mir-1307, hsa-mir-338, hsa-mir-93, hsa-mir-2355, hsa-mir-16-2, hsa-mir-15b, hsa-mir-210, hsa-mir-130b

## Data Availability

The raw data for ROSMAP are available at https://adknowledgeportal.synapse.org/, accessed on 27 October 2025. The raw data for BRCA, LGG, and KIPAN are available at https://portal.gdc.cancer.gov/, accessed on 27 October 2025. Code for this study is available from the corresponding author upon reasonable request.

## Supplementary Materials

The following supporting information can be downloaded at https://www.mdpi.com/article/10.3390/biology14121764/s1, Table S1: Performance comparison results for different omics combinations; Table S2: Ablation experimental for KIPAN and LGG; Table S3: Performance of different graph structure configurations for KIPAN and LGG; Table S4: Graph layer depth experiment; Table S5: Cross-Omics fusion mechanism comparison for KIPAN and LGG; Table S6: Biomarkers identified for BRCA; Table S7: Enrichment result; Table S8: Protein–Protein Interaction network; Table S9: Hub genes result; Table S10: Drug-Gene Interactions.

## Abbreviations

The following abbreviations are used in this manuscript:

GCN	Graph Convolutional Network
GAT	Graph Attention Network
TCPs	True-Class Probabilities
OLA	Omics-Linked Attention
BRCA	Breast Cancer
KIPAN	Kidney Cancer
ROSMAP	Alzheimer’s Disease
LGG	Lower-Grade Glioma
