# Supplementary material for: A Graph-Based Deep Learning Framework with Gating and Omics-Linked Attention for Multi-Omics Integration and Biomarker Discovery
Source: Biology (Basel). 2025 Dec 10;14(12):1764. doi: 10.3390/biology14121764 (PMC12731160; doi:10.3390/biology14121764)
Supplement: Supplementary file 1 [file biology-14-01764-s001.zip › (Table S1-S5)Supplementary Material.pdf]

Supplementary Material: A Graph-Based Deep Learning Framework with Gating and Omics-Linked Attention for Multi-Omics Integration and Biomarker Discovery

Dataset	Omics	metrics	MOGONET	MMDynamics	CLCLSA	MOSGAT	Ours
BRCA	mRNA+ meth+ miRNA	ACC	0.821±0.007	0.865±0.007	0.848±0.010	0.873±0.004	**0.895±0.001**
		F1_weighted	0.730±0.012	0.868±0.007	0.8451l±0.015	0.878±0.003	**0.899±0.003**
		F1_marco	0.776±0.007	0.658±0.023	0.772±0.037	0.847±0.005	**0.878±0.006**
	mRNA+ meth	ACC	0.821±0.007	0.879±0.003	0.851±0.012	0.874±0.003	**0.891±0.003**
		F1_weighted	0.813±0.007	0.883±0.003	0.847±0.016	0.878±0.003	**0.895±0.003**
		F1_marco	0.776±0.007	0.853±0.008	0.773±0.031	0.845±0.007	**0.876±0.008**
	mRNA+ miRNA	ACC	0.710±0.006	0.814±0.005	0.763±0.007	0.803±0.009	**0.827±0.005**
		F1_weighted	0.660±0.017	0.813±0.005	0.733±0.008	0.802±0.007	**0.852±0.041**
		F1_marco	0.492±0.028	0.767±0.004	0.593±0.015	0.756±0.006	**0.802±0.009**
	meth+ miRNA	ACC	0.712±0.010	0.804±0.009	0.766±0.006	0.800±0.005	**0.831±0.005**
		F1_weighted	0.662±0.021	0.802±0.010	0.739±0.009	0.800±0.007	**0.834±0.005**
		F1_marco	0.506±0.039	0.753±0.016	0.602±0.024	0.753±0.009	**0.801±0.010**
KIPAN	mRNA+ meth+ miRNA	ACC	0.997±0.005	**0.999±0.002**	**0.999±0.002**	**0.999±0.002**	**0.999±0.002**
		F1_weighted	0.997±0.005	**0.999±0.002**	**0.999±0.002**	**0.999±0.002**	**0.999±0.002**
		F1_marco	0.995±0.010	**0.999±0.002**	**0.999±0.002**	**0.999±0.002**	**0.999±0.002**
	mRNA+ meth	ACC	0.906±0.057	0.957±0.007	0.998±0.002	**0.999±0.002**	**0.999±0.002**
		F1_weighted	0.886±0.063	0.954±0.008	0.998±0.002	**0.999±0.002**	**0.999±0.002**
		F1_marco	0.787±0.122	0.912±0.012	0.997±0.004	**0.999±0.002**	**0.999±0.002**
	mRNA+ miRNA	ACC	0.923±0.029	0.961±0.004	0.970±0.000	0.975±0.004	**0.978±0.002**
		F1_weighted	0.899±0.048	0.958±0.004	0.968±0.000	0.973±0.004	**0.978±0.003**
		F1_marco	0.765±0.130	0.917±0.008	0.939±0.000	0.958±0.008	**0.967±0.005**
	meth+ miRNA	ACC	0.979±0.015	0.840±0.068	**0.999±0.002**	0.995±0.000	0.997±0.002
		F1_weighted	0.978±0.015	0.802±0.010	**0.999±0.002**	0.995±0.000	0.997±0.002
		F1_marco	0.965±0.023	0.753±0.016	**0.999±0.002**	0.992±0.003	0.998±0.001
RSOMAP	mRNA+ meth+ miRNA	ACC	0.825±0.004	0.821±0.018	0.824±0.008	0.830±0.012	**0.879±0.004**
		F1	0.821±0.006	0.827±0.015	0.831±0.009	0.830±0.012	**0.879±0.004**
		AUC	0.884±0.007	0.901±0.004	0.901±0.007	0.873±0.011	**0.903±0.006**
	mRNA+ meth	ACC	0.800±0.011	0.823±0.004	0.787±0.009	0.810±0.009	**0.826±0.004**
		F1	0.799±0.011	0.823±0.004	0.794±0.008	0.809±0.009	**0.826±0.004**
		AUC	0.830±0.015	0.870±0.003	0.860±0.007	0.859±0.014	**0.872±0.007**
	mRNA+ miRNA	ACC	0.751±0.032	0.836±0.013	0.792±0.013	0.823±0.014	**0.842±0.007**
		F1	0.745±0.036	0.836±0.013	0.798±0.015	0.822±0.015	**0.842±0.007**
		AUC	0.791±0.028	0.892±0.007	0.858±0.003	0.884±0.014	**0.898±0.005**
	meth+ miRNA	ACC	0.707±0.019	0.749±0.008	0.689±0.022	0.710±0.021	**0.749±0.005**
		F1	0.690±0.014	0.747±0.009	0.721±0.022	0.710±0.021	**0.749±0.004**
		AUC	0.711±0.015	**0.810±0.018**	0.723±0.018	0.750±0.022	0.788±0.011
LGG	mRNA+	ACC	0.834±0.008	0.814±0.008	0.841±0.003	0.824±0.007	**0.867±0.005**
	meth+	F1	0.834±0.008	0.824±0.007	0.841±0.004	0.823±0.007	**0.867±0.005**

	miRNA	AUC	0.867±0.013	0.862±0.003	0.892±0.003	0.857±0.013	**0.894±0.009**
	mRNA+ meth	ACC	0.821±0.017	0.845±0.007	**0.850±0.008**	0.801±0.005	0.808±0.003
		F1	0.820±0.018	0.844±0.008	**0.851±0.008**	0.800±0.007	0.808±0.003
		AUC	0.831±0.031	**0.891±0.005**	0.889±0.003	0.826±0.008	0.853±0.010
	mRNA+ miRNA	ACC	0.793±0.027	0.848±0.005	0.847±0.007	0.803±0.009	**0.862±0.003**
		F1	0.798±0.036	0.848±0.005	0.847±0.008	0.802±0.007	**0.862±0.003**
		AUC	0.810±0.036	0.891±0.007	**0.894±0.002**	0.756±0.006	0.886±0.003
	meth+ miRNA	ACC	0.769±0.025	0.807±0.010	0.814±0.003	0.816±0.006	**0.841±0.005**
		F1	0.774±0.029	0.806±0.010	0.818±0.002	0.815±0.006	**0.841±0.005**
		AUC	0.731±0.047	0.867±0.004	0.865±0.005	0.850±0.009	**0.876±0.010**

S.1 Performance comparison experiment results of different omics combinations.

Dataset	metrics	No GSL	No GC	No OLA	No Ablation
KIPAN	ACC	0.976±0.003	0.994±0.002	0.998±0.002	**0.999±0.002**
	F1_weighted	0.975±0.004	0.996±0.002	0.998±0.002	**0.999±0.002**
	F1_macro	0.963±0.006	0.997±0.001	0.998±0.001	**0.999±0.002**
LGG	ACC	0.826±0.007	0.864±0.005	0.855±0.008	**0.867±0.005**
	F1	0.825±0.007	0.864±0.005	0.855±0.008	**0.867±0.005**
	AUC	0.852±0.014	0.875±0.006	**0.895±0.004**	0.894±0.009

S.2 Ablation experimental with KIPAN and LGG.

Dataset	metrics	GAT-GAT-GCN	GAT-GCN-GAT	GAT-GCN-GCN	GCN-GAT-GAT	GCN-GAT-GCN
KIPAN	ACC	0.997±0.002	0.999±0.002	0.994±0.006	0.998±0.002	**0.999±0.002**
	F1_weighted	0.997±0.002	0.999±0.002	0.994±0.006	0.998±0.002	**0.999±0.002**
	F1_macro	0.997±0.001	0.999±0.002	0.994±0.006	0.998±0.001	**0.999±0.002**
LGG	ACC	0.859±0.013	0.840±0.005	0.837±0.011	0.856±0.012	**0.867±0.005**
	F1	0.859±0.013	0.839±0.006	0.836±0.011	0.856±0.012	**0.867±0.005**
	AUC	0.880±0.013	0.866±0.008	0.863±0.008	**0.894±0.005**	0.894±0.009

S.3 Performance of different graph structure configurations with KIPAN and LGG.

Dataset	metrics	GCN-GAT	GCN-GAT-GCN	GCN-GAT-GCN-GAT
BRCA	ACC	0.892±0.002	**0.895±0.001**	0.507±0.202
	F1_weighted	0.896±0.002	**0.899±0.003**	0.414±0.224
	F1_macro	0.877±0.005	**0.878±0.006**	0.285±0.187
ROSMAP	ACC	0.864±0.006	**0.879±0.004**	0.741±0.115
	F1	0.864±0.006	**0.879±0.004**	0.705±0.178
	AUC	0.884±0.004	**0.903±0.006**	0.735±0.189

Dataset	metrics	GCN-GAT	GCN-GAT-GCN	GCN-GAT-GCN-GAT
KIPAN	ACC	**0.999±0.002**	**0.999±0.002**	0.618±0.294
	F1_weighted	**0.999±0.002**	**0.999±0.002**	0.543±0.321
	F1_macro	**0.999±0.002**	**0.999±0.002**	0.409±0.226
LGG	ACC	0.864±0.006	**0.867±0.005**	0.741±0.115
	F1	0.864±0.006	**0.867±0.005**	0.705±0.178
	AUC	0.884±0.004	**0.894±0.009**	0.735±0.189

S.4 Graph layer depth Experiment.

Dataset	metrics	Replace VCDN	Replace self-attention	No Ablation
KIPAN	ACC	0.995±0.001	0.998±0.002	**0.999±0.002**
	F1_weighted	0.995±0.001	0.998±0.002	**0.999±0.002**
	F1_macro	0.996±0.001	0.998±0.001	**0.999±0.002**
LGG	ACC	0.847±0.018	0.862±0.003	**0.867±0.005**
	F1	0.847±0.018	0.862±0.003	**0.867±0.005**
	AUC	0.878±0.018	0.881±0.010	**0.894±0.009**

S.5 Cross-Omics fusion mechanism comparison with KIPAN and LGG.
